# Supplementary material for: Pretreatment of Melanoma Cells with Aqueous Ethanol Extract from Madhuca longifolia Bark Strongly Potentiates the Activity of a Low Dose of Dacarbazine
Source: Int J Mol Sci. 2024 Jun 29;25(13):7220. doi: 10.3390/ijms25137220 (PMC11241726; doi:10.3390/ijms25137220)
Supplement: Supplementary file 1 [file ijms-25-07220-s001.zip › ijms-3050060-supplementary.pdf]

Supplementary Materials:

Figure S1. MS and MS/MS spectra of the investigated peaks obtained for MLE.

Peak (1)

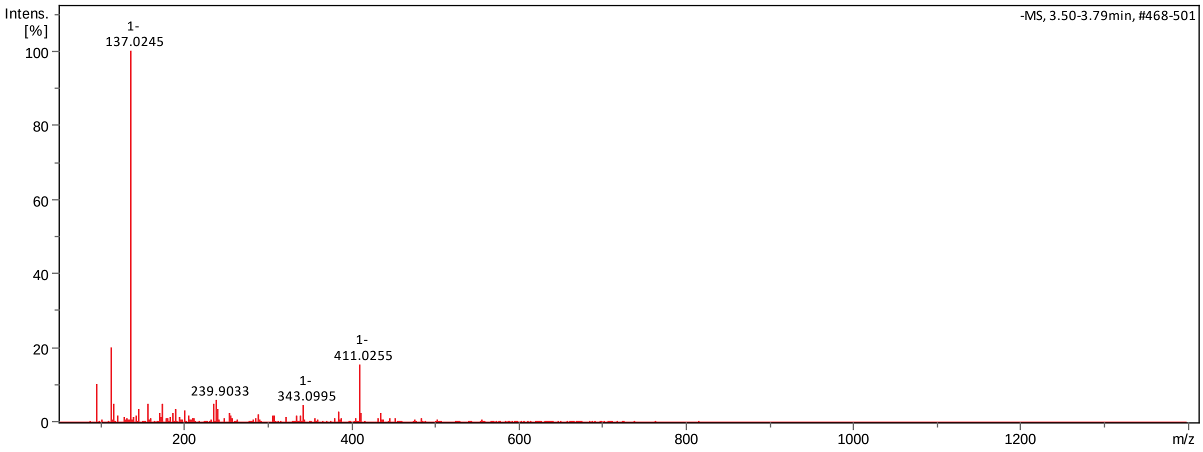

Peak (2)

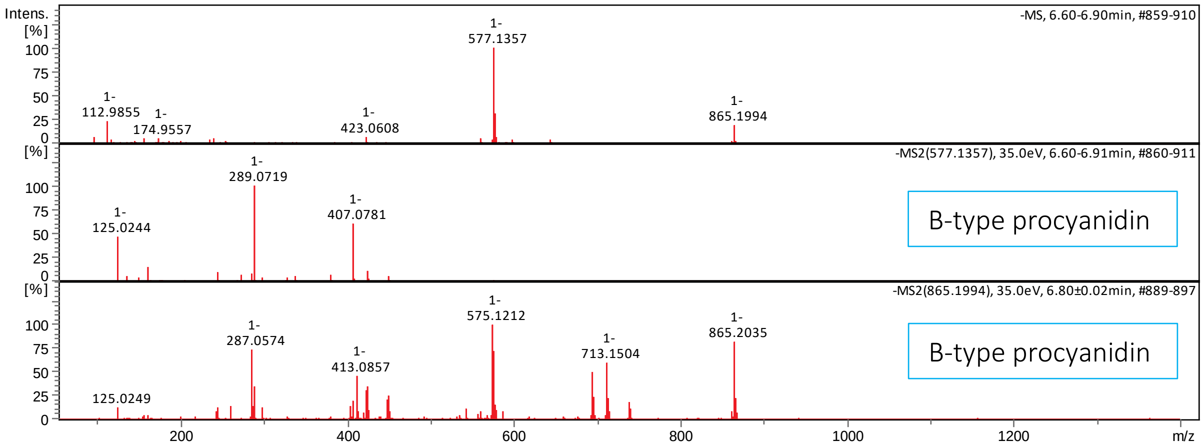

Peak (3)

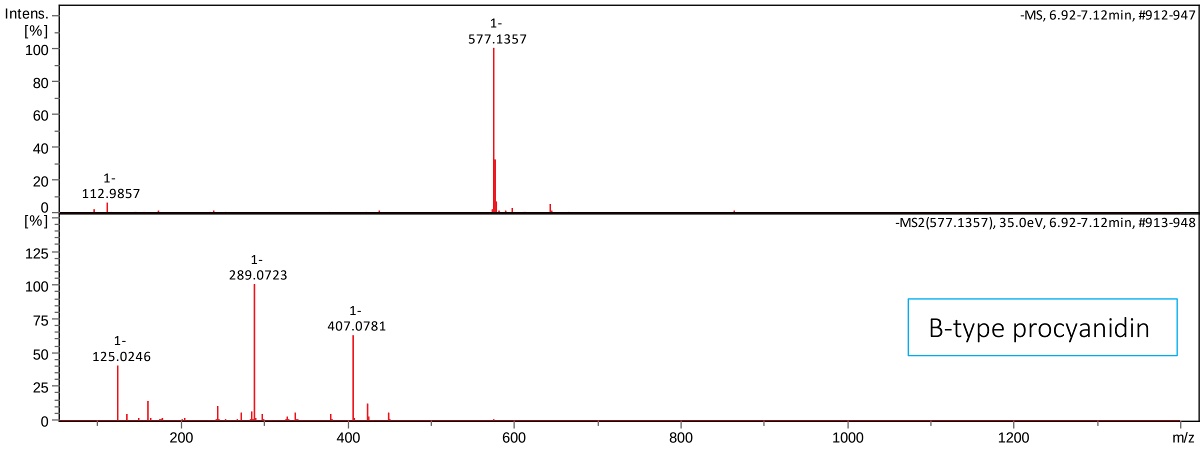

Peak (4)

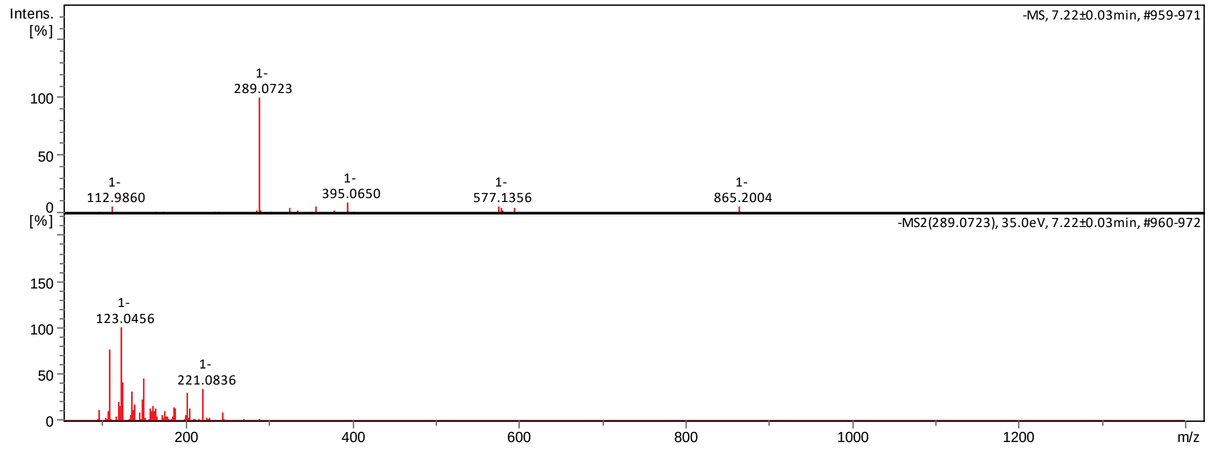
Intens.
[%]
-MS, 7.22±0.03min, #959-971
1-
289.0723
1-
112.9860
1-
395.0650
1-
577.1356
1-
865.2004
-MS2(289.0723), 35.0eV, 7.22±0.03min, #960-972
1-
123.0456
1-
221.0836
m/z

Peak (5)

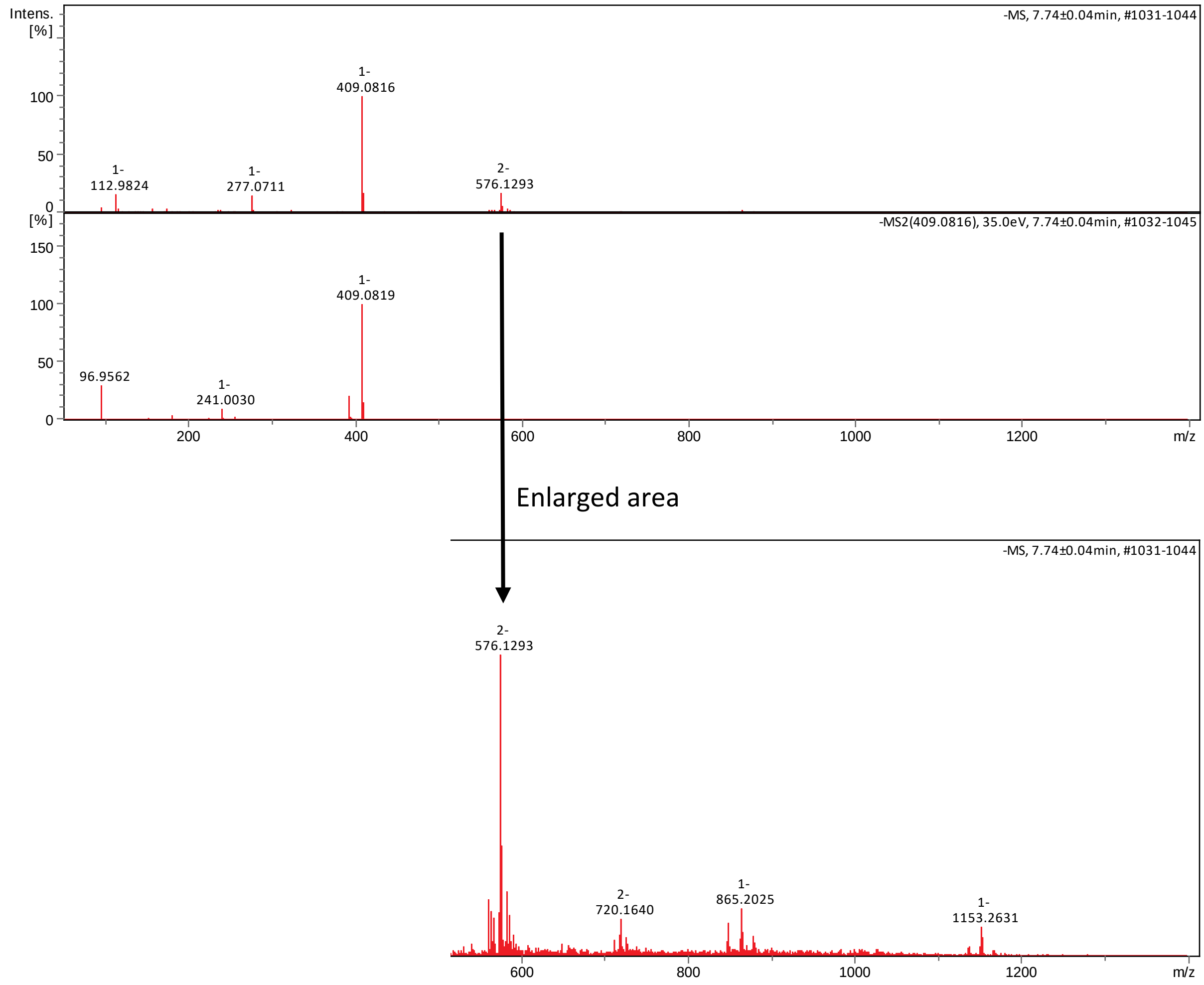
Intens.
[%]
-MS, 7.74±0.04min, #1031-1044
1-
409.0816
1-
112.9824
1-
277.0711
2-
576.1293
-MS2(409.0816), 35.0eV, 7.74±0.04min, #1032-1045
1-
409.0819
96.9562
1-
241.0030
m/z
Enlarged area
-MS, 7.74±0.04min, #1031-1044
2-
576.1293
2-
720.1640
1-
865.2025
1-
1153.2631
m/z

Peak (6)

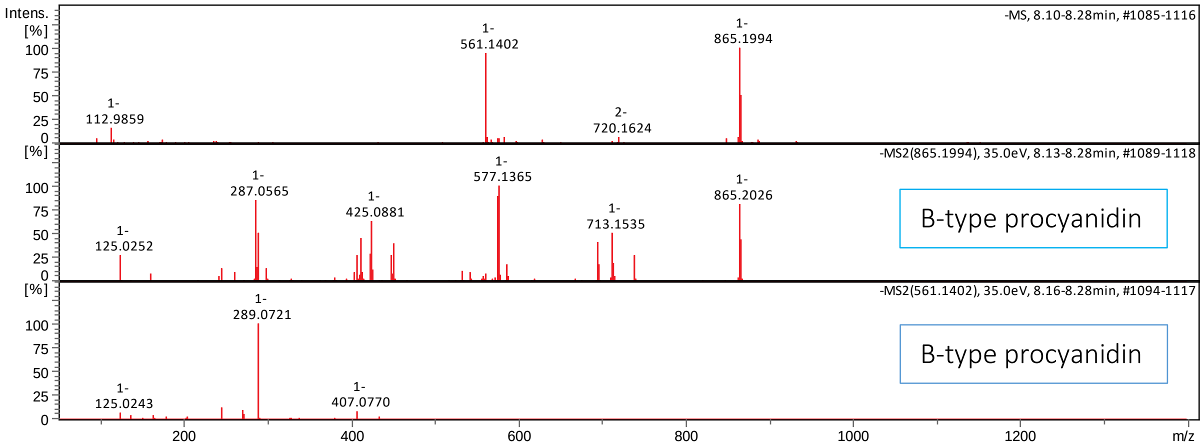

Peak (7)

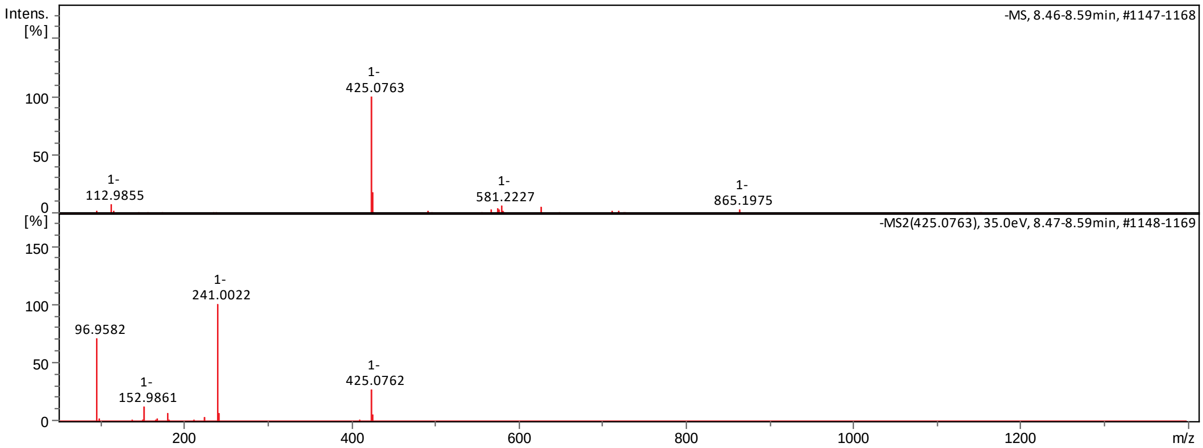

Peak (8)

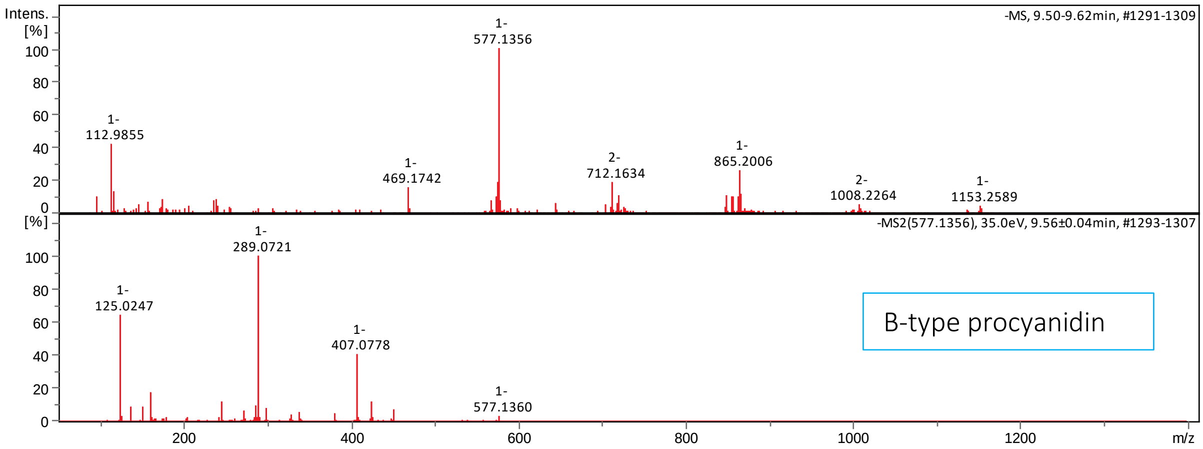

Peak (9)

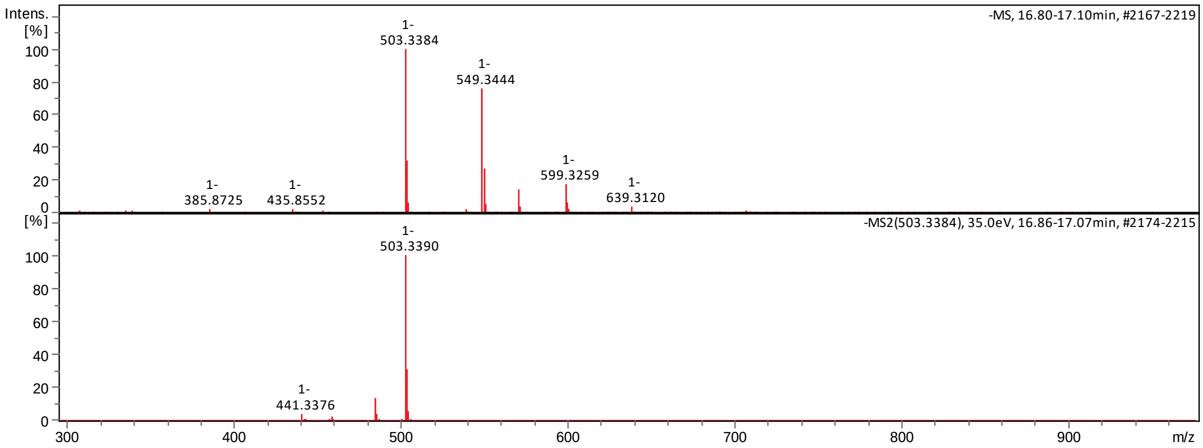
Intens.
[%]
-MS, 16.80-17.10min, #2167-2219
1-
503.3384
1-
549.3444
1-
599.3259
1-
639.3120
1-
385.8725
1-
435.8552
-MS2(503.3384), 35.0eV, 16.86-17.07min, #2174-2215
1-
503.3390
1-
441.3376
m/z
